# Prospective longitudinal cohort of Argentinean patients with progressive supranuclear palsy and corticobasal syndrome: A platform for epidemiological and translational research

**DOI:** 10.1016/j.prdoa.2025.100339

**Published:** 2025-05-15

**Authors:** Maria Jose Angel, Gabril F. Mizraji, Gonzalo Gomez-Arevalo, Garcia Silvia, Maria Eugenia Gonzalez-Toledo, Avale Elena, Paez-Paz Indiana, Falzone Tomas, Holubiec Mariana, C. Peralta Maria, Castano Federico, Sevlever Gustavo, Mezmezian Monica, Marras Connie, Gershanik Oscar, Chade Anabel, Pardo Carolina, Rellan Florencia, Campastri Ana, Couto Blas

**Affiliations:** a Institute of Cognitive and Translational Neuroscience (INCyT) Favaloro-INECO-CONICET, Buenos Aires, Argentina; b Instituto de Nacional de Geriatría, Santiago de Chile, Chile; c Unidad de Movimientos Anormales, Instituto de Neurociencias de la Fundación Favaloro, Buenos Aires, Argentina; d INGEBI, Institute of Investigation in Genetic Engineering and Molecular Biology Dr Héctor N Torres, Argentina; e Instituto de Biología Celular y Neurociencias (IBCN), Universidad de Buenos Aires, CONICET-UBA, Instituto de Investigación en Biomedicina de Buenos Aires (IBioBA), Partner Institute of the Max Planck Society, CONICET-MPSP, Buenos Aires, Argentina; f Clínica de Enfermedad de Parkinson y Movimientos Anormales, Departamento de Neurociencias, Hospital Universitario CEMIC, Centro de Educación Médica e Investigaciones Clínicas “Norberto Quirno”, Buenos Aires, Argentina; g Laboratorio de Neuropatología y Biología Molecular, Banco de cerebros, Fleni, Argentina; h Edmond J. Safra Program in Parkinson’s Disease, Rossy Program for PSP Research and the Morton and Gloria Shulman Movement Disorders Clinic, Toronto Western Hospital, Toronto, ON, Canada; i Institute of Cognitive Neurology (INECO), Buenos Aires, Argentina

**Keywords:** Progressive supranuclear palsy, Corticobasal syndrome, Biobank, Environmental, Longitudinal cohort

## Abstract

**Background:**

Progressive supranuclear palsy (PSP) and corticobasal syndrome (CBS) are neurodegenerative disorders with disabling postural instability, falls and cognitive impairment associated with 4-repeat tau protein neuropathology. The low prevalence (6/100,000 inhabitants) is estimated with data only from North American, European countries or Japan.

**Objective:**

A multicenter initiative for studying the epidemiology and tau biology of Argentinean patients with PSP and CBS and their ovelaps, Consorcio Argentino de Investigación Traslacional en Tauopatias Primarias (CAITauP).

**Methods:**

A prospective observational cohort of people with PSP (PwPSP), CBS and PSP/CBS overlaps will be longitudinally studied aiming at recruitment of 75 patients in 3 years. Deep clinical phenotyping, neurocognitive testing every 12 months, and biosamples collection at baseline are planned. Disease severity will be assessed every 6 months and history of environmental exposures will be assessed at baseline.

**Results:**

33/75 expected patients with PSP (19) and CBS (14) have been enrolled. Female gender is 56%, mean age 76, with estimated disease duration of 4.73 years. More than 60% of enrolled patients have completed first visit and biosamples collection, one patient underwent brain donation, pathological diagnosis of PSP, clinical Richardson syndrome. Environmental exposures of a preliminary subsample are reported.

**Conclusions:**

The CAITauP enrolled a cohort of Argentinean PwPSP for deep clinical phenotyping and biobanking. Despite the rarity of PSP/CBS, we highlight the feasibility of establishing local cohorts in developing countries for translational research. The consortium aims to address underrepresented groups of Argentinean to improve diagnosis, treatment, and to create trial-ready cohorts.

## 1 Introduction

Progressive supranuclear palsy (PSP) and corticobasal degeneration are primary tauopathies with a world-wide prevalence of 6/100,000 mostly estimated from studies including patients from Europe, North America and Japan, highlighting the underrepresentation of South American populations in the research of primary tauopathies, with only one single study published from Brazil [1].

Argentinean populations share a unique genetic admixture resulting from historical and cultural interactions of three ancestral sources: i) original inhabitants, with genetic lineages originated from a single migration from Asia around 15,000–20,000 years ago [2]; ii) European genetic influence beginning with 15th century colonization (Spain and Portugal, followed by later waves of immigrants from Italy, Germany, and Eastern Europe); and iii) African Ancestry, that arose during the transatlantic slave trade. Genomic-Wide Association (GWAS) studies revealed regional admixture patterns that vary widely across South America. African genetic contributions are particularly pronounced in countries like Brazil, Colombia, and Venezuela, where large populations of African descent reside. While European ancestry dominates in countries like Argentina, Uruguay, and southern Brazil, often exceeding 70% in urban and coastal areas [3].

On the other hand, patients from South America share lifestyle habits, including the consumption of regional beverages derived from yerba-mate (*Ilex paraguariensis),* which contains compounds such as chlorogenic acid and theobromine, with demonstrated neuroprotective effect in dopaminergic neurons [1,[4], [5], [6], [7]]. People from tropical regions also consume chirimoya, an *annonaceae* fruit containing several neurotoxic substances such as annonacin, which induce tau aggregation [8]. Argentinean and the South American population has multiple clusters of rural inhabitants exposed to known risk factors for PSP such as well water and pesticides [9]. Altogether, these compose a specific profile of environmental exposure that might account for a different vulnerability in these populations to develop neurodegenerative diseases including 4-repeat tauopathies [[10], [11], [12]]. The establishment of biobanks containing fluid and tissue samples from patients with PSP is crucial for advancing our understanding of this diseases. Due to the lack of diversity in the research cohorts generated so far it is unclear if results are generalizable to other populations and may impede the development of effective, and inclusive therapeutic strategies. Genetic risk factors for neurodegenerative disorders vary significantly across different populations, underscoring the need for more inclusive genetic studies [[13], [14], [15], [16], [17]] which is supported by the challenges of translation of polygenic risk scores across populations with admixed ancestries [18]. A South American biobank would facilitate the identification of unique genetic variants and environmental interactions that may influence disease onset and progression in these groups. This approach will enhance the inclusivity and applicability of research findings.

There is evidence of geographical, toxic, and racial/ethnic influences on the risk for PSP and other atypical parkinsonism that may relate to the presence of genetic modifiers of the disease course and to environmental-genetic interactions not yet clarified [14,15]. In this context, the study of pathogenic implications of genetic-environmental factors in the onset and progression of primary tauopathies will benefit from studies including data from highly admixed, underrepresented populations like South American patients with PSP (PwPSP).

With the goal of filling those gaps, a Consortium for Research of Primary Tauopathies (Consorcio Argentino de Investigación en Taupatias Primarias—CAITauP, https://ingebi-conicet.gov.ar/caitaup/) has been created to collect clinical information and peripheral tissue biosamples to develop a longitudinal cohort and in vitro human models of the disease in which to test putative therapeutic strategies. We report here the design of the first longitudinal clinical, imaging and biological study of PwPSP and CBS in Argentina from the CAITauP.

## 2 Materials and methods

### 2.1 Study design and clinical data collection

**Fig. 1:**
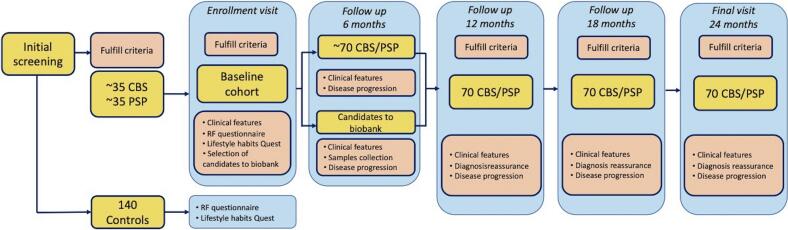
Design and flow of patient visits through the CAITauP initiative in Argentina. * *HC were only recruited for the purpose of comparison in the sub-study of environmental exposures.*

The study has a prospective observational design and includes the deep clinical phenotyping and longitudinal follow up of a cohort of PwPSP (Fig. 1). The inclusion criteria for patients are insidious onset of symptoms at age 40 years or older; fulfillment of the movement disorders society (MDS)-endorsed criteria for possible or probable diagnosis of PSP [19], and/or CBS [20].

Data collection is harmonized with other international registries and a specific digitized protocol with all instruments in Spanish has been developed within Redcap platform. During the enrollment visit and every six-months patients are evaluated by neurology specialists trained in movement disorders (Fig. 1). Physical and neurological exam is recorded by video and based on the structured examen proposed by the Alliance for Research in Frontotemporal Lobar Degeneration (ARFTLD) and the Rossy Centre for PSP in Toronto [21]. Neurocognitive status is assessed at baseline and yearly re-assessed using a standardized cognitive battery developed and reviewed by cognitive neurology and neuropsychology specialists. The battery includes screening test, Montreal Cognitive Assessment (MOCA) [22], Frontal Assessment Battery [23], INECO frontal screening test (IFS) [24], and the Toronto Cognitive Assessment (TorCA) [25] as well as specific tests for each cognitive domain: memory with the Rey Auditory and Visual Verbal Learning tests [26]; Trail-making test A/B; direct/inverse digit-span [26]; phonological/semantic fluencies [27]; Wisconsin Card Sorting Test [28].

Behavioral changes and neuropsychiatric symptoms will be evaluated by questionnaires and scales including geriatric depression, anxiety, apathy, anosognosia, Zarit-caregiver burden [29]. Additionally, the non-motor symptoms questionnaire for Parkinson’s Disease (PD) [30], and other functionality scales and questionnaires for quality of life [31] related to neurological health and parkinsonism (Table 1). Instruments are to be completed by examiners, clinical raters in RedCAP, and auto-administered scales are sent to the patient/caregiver via email to be filled out online.

**Table 1:** Instruments and questionnaires applied to enrolled PwPSP in the CAITau.

	**Baseline**	**Visit 1** **1**–**3 mo.**	**Visit 2** **6 mo.**	**Visit 3** **12 mo.**	**Visit 4** **18 mo.**	**Visit 5** **24 mo.**
Follow-up history intake form	_X_		_X_	_X_	_X_	_X_
Health history assessment	_X_					
Full neurological exam	_X_		_X_		_X_	_X_
Neurological exam: videorecording		_X_				_X_
Focused neurological exam		_X_		_X_		
PSP clinical features	_X_		_X_	_X_	_X_	_X_
MDS-UPDRS I-IV	_X_		_X_	_X_	_X_	_X_
PSP Rating scale	_X_		_X_	_X_	_X_	_X_
MOCA	_X_		_X_		_X_	
NMSS	_X_					
PDQ-39	_X_					
Demographics − Family history	_X_		_X_			
Geriatric depression rating scale	_X_		_X_			_X_
Hamilton anxiety scale	_X_					
Schwab and England scale	_X_					
RFQ lifestyle	_X_					
RFQ Pesticides	_X_					
RFQ assesses exposures	_X_					
Neuropsychological Testing		_X_		_X_		_X_
Apathy (AES)		_X_		_X_		
Frontal System (FrSBE)		_X_		_X_		
Impulsivity (BIS II)		_X_		_X_		
Behavior (CBI)		_X_		_X_		
Samples collection for biobank		_X_				

mo., months; NMSS, Non-Motor Symptoms Scale; PDQ-39, Parkinson’s Disease quality of life Questionnaire; RFQ, Risk Factor Questionnaire; FrSBE, Frontal Systems Behavior Scale; BIS-11, Barratt Impulsiveness Scale; CBI, Cambridge Behavioural Inventory.

Healthy control participants are recruited only for the purpose of comparison in the sub-study of environmental exposures. The rationale behind this is to apply the RFQ questionnaire originally designed for PD environmental risk factors, and with a case-control design we also included HC. Enrollment criteria for HC are age older than 40-years, without a diagnosis of PSP/CBS, Parkinson disease, AD, other neurodegenerative disease, or chronic psychiatric disorders of moderate to severe severity. The sources of recruitment for HC consist of the main caregiver (spouse or sibling) of the patient if they qualify. PD patients are enrolled during consecutive follow-up visits to the outpatient movement disorders clinic solely for comparison purposes in the environmental exposures sub-study.

### 2.2 Environmental and lifestyle data collection

A self-administered questionnaire, the Risk Factors Questionnaire [32], will be used to screen for environmental exposures at work and in areas of residence, family history of PD and Alzheimer’s Disease (AD), and consumption habits of coffee, tea, alcohol. This instrument also screens for women’s health information, which has been previously related to PSP [33,34]. We have included as well an *ad hoc* designed questionnaire to screen for chirimoya (*annonaceae*) fruit, and for consumption of yerba mate based on previous reports [4,35].

### 2.3 Biosamples data collection

At baseline, all patients are offered to participate in biosamples collection for translational research that include peripheral blood, skin biopsy and cerebrospinal fluid (CSF; see Fig. 1). A registered nurse draws 20 mL of blood then processed for DNA extraction and proteomic analyses. A dermatology specialist (A.Ca.) performs three 4-mm skin punch biopsies for the generation of primary fibroblast culture [36] and for further study using seeding-amplification assays for tau and alpha-synuclein.

In terms of neuroimaging, brain scans will be performed to enrolled patients with brain MRI, ^18^Flurodeoxyglucose-PET, and ^18^Flurodopa-PET in the CEMIC institute of Buenos Aires. The scanning protocol and funding are under revision.

Participation in a brain donation program is also offered. Autopsy brain material is processed for standard immunohistochemistry and frozen fixed for further molecular characterization of RNA and protein expression levels.

### 2.4 Recruiting strategies and promotion

Patient recruitment comprises multiple sources: i) consecutive patients seen at the Institute of Cognitive Neurology of Buenos Aires, and at the Movement Disorders Unit of the Favaloro Foundation University Hospital of Buenos Aires; ii) referral from additional neurology clinics across Argentina: in Buenos Aires, Salta, Tucuman, Cordoba, Cipolletti, and Trelew; iii) additional cases will be included from the National Registry for PSP-CBS endorsed by the Movement Disorders Study Group of the Argentinean Neurological Society. Early during the design, a survey among members of that group evidenced 22 specialists actively following at least one and up to three cases interested in participating. Several promotional activities have been organized and are planned to foster referrals including: the Argentinean Neurological Society (ANS) annual meeting was held to launch the initiative and present translational goals of research; clinical case webinars organized by the ANS with the Pan-American Section of the MDS; smaller case presentation rounds with Argentinean neurologists were organized through 2023–2024.

The protocol has been approved by the Board on Human Ethics of the University of Buenos Aires (2024–01311609-UBA-DMEA#FMED), and the procedures follow the Helsinki guidelines of research with humans.

Statistical analysis using the Student’s *t*-test for independent samples to compare continuous variables between two groups, Mann-Whitney’s U for ordinal variables, and associations with the Spearman ρ and further multiple linear regressions in STATA software.

## 3 Results

### 3.1 Clinical preliminary data

**Fig. 2:**
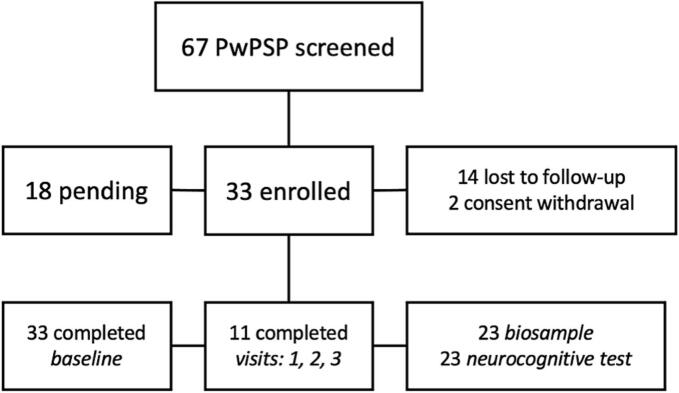
Screening and enrollment status of the CAITauP as of October 2024.

Between November 2022 and October 2024, a total of 67 patients were screened, with 33 of them enrolled with clinical diagnosis of PSP or CBS, 18 pending baseline visits (all of them included in Table 2), and 14 loss-of-follow up (20%), and four who refused participation (Fig. 2). About 30% of patients have completed up to visit-3 (18 months follow up), and over 70% of them completed biosamples collection and neurocognitive assessment. The mean age at enrolment was 76 (range 67–88) years, 56% were female sex, mean disease duration since symptom onset 4.73 (range 1–11) years. PSP-RS was the most common clinical presentation (31 patients), although also common are corticobasal syndrome and progressive-gait freezing (PGF)-predominant variant PSP. Mean disease severity was 49 points in PSPRS (Table 2).

**Table 2:** Demographic and clinical features of PwPSP and CBS enrolled in the CAITauP.

Age, Mean(Range)	76 (67–88)
Sex, Female %	56
Duration Symptoms, Mean(Range)	4.73(1–11)
Main PSP-predominant variant	*n*
Richardson Syndrome	31
PSP-Corticobasal Syndrome*	7
Progressive Gait Freezing	3
PSP-Parkinsonism	2
Frontal	0
Speech/Language	1
Corticobasal Syndrome, Total	14
Isolated Corticobasal Syndrome	7
Severity, Mean PSPRS	49
Family History of Parkinson, %Positive	13
Family History of Alzheimer's, %Positive	14

* *All meet criteria for PSP-predominant corticobasal syndrome by MDS-PSP criteria.*

### 3.2 Environmental and lifestyle

We collected information on environmental exposures from the Risk Factor Questionnaire and found a low prevalence of rural residence (5 patients), one patient lived in a house next to agricultural fields during early life but reported no direct exposure to pesticides. The reported frequency of pesticide exposure was 14.25%; and 37% of PSP patients reported exposure to well water. The weekly consumption of caffeine in PwPSP was 26 cups, represented by the ingestion of coffee, black tea, and mate. The frequency of smoking was 53%.

### 3.3 Biobank results

Skin biopsies from 24 PwPSP were collected (70% of enrolled patients), in addition to biopsies from four aged-matched controls. To date, a total of 16 fibroblast cultures from patients and 2 controls have persisted with a significant amplification rate. For each successfully grown culture eight vials containing two hundred thousand cells were cryopreserved and are available for further amplification or reprograming. Fibroblast identity was confirmed through morphology analysis as well as positive Vimentin immunostaining. The cultures originating from tissue from PwPSP with positive family history or carrying identifiable genetic mutations will be selected for reprogramming into iPSC, followed by the development of in vitro neuronal models.

A 100% of the participants agreed to be enrolled in the CurePSP Genetic Registry for analyses of Genome-wide sequencing. In terms of neuropathology, two cases have been processed after brain donation with a report of typical PSP pathology in both, one of which genotyping profiles will be reported in a separate publication.

## 4 Discussion

This article presents the design of the CAITauP initiative and initial longitudinal cohort of PwPSP in Argentina. The strengths of the cohort are its prospective design and breadth of data collection, harmonized with current similar international endeavors. In 25 months, 44% patients enrolled have completed a first follow-up visit. By applying the most recent criteria for diagnosis of PSP and deep phenotyping of the patients, the CAITauP will help increase the representation in research of the clinical variants of PSP. This is important since there is a strong bias towards over-representation of the Richardson’s syndrome (PSP-RS) in previous clinical and clinical-pathological studies considering that RS is the most prevalent and with highest positive predictive value [37,38]. We expect that as the general awareness of this platform for epidemiological and translational research spreads within the local neurological community, referral delay will decrease, and the number of more rare clinical variants will increase. The low rate of screened/enrolled patients in CAITauP is attributed to PwPSP in advanced stages that has difficulted access to the clinic or caused lost to follow-up. This is expected and in line with a shorter survival (7.8 years) [9,39] and a delay in diagnosis/referral of about 5.6 years in PwPSP (current data). The educational and promoting activities organized by the CAITauP will further the local clinical recognition of PwPSP and hopefully decrease the diagnostic delay in our communities.

Genetic investigations of cases have been prioritized during the first 2 years, with referral to the Cure PSP Genetic Registry and GP2 global initiative. The first batch of genome-wide sequencing from PwPSP from CAITauP will be reported soon. Recognition of local genetic variants associated with PSP will guide the production of iPSC-neuron models for the studying the biology of PwPSP not done before in Argentina for this progressive and inevitably fatal neurodegenerative diseases.

The longitudinally collected data of the CAITauP will allow the creation of trial-ready cohorts of patients to be fast-tracked to participate in therapeutic trials. Regarding neuroimaging and neuropathology, the brain imaging protocol is set and starting soon whereas we plan to enlarge the visibility of the brain donation program hoping to increase awareness and the number of the donations. These will allow comparative studies with other cohorts, providing invaluable resources for tissue-based research in PSP and new insights into diagnosis and management.

## Disclosures and competing interests

Funding is received from a Cure PSP Foundation Grant (Award ID: 684-2023-06-Pathway), from Facultad de Medicina, Universidad de Buenos Aires (UBACYT-2023- 20020220200095BA), and PI (B.C.) has received a Clinician Scientist Fellowship-in Diversity Award from the Alzheimer’s Association.

## CRediT authorship contribution statement

**Angel Maria Jose:** Writing – original draft, Methodology, Investigation, Formal analysis, Data curation. **Mizraji Gabril F.:** Writing – review & editing, Methodology, Data curation. **Gomez-Arevalo Gonzalo:** Data curation. **Garcia Silvia:** Data curation. **Gonzalez-Toledo Maria Eugenia:** Data curation. **Avale Elena:** Writing – review & editing, Methodology, Investigation, Formal analysis, Data curation, Conceptualization. **Paez-Paz Indiana:** Data curation. **Falzone Tomas:** Writing – review & editing, Methodology, Investigation, Formal analysis, Data curation, Conceptualization. **Holubiec Mariana:** Data curation. **C. Peralta Maria:** Writing – review & editing, Methodology, Data curation. **Castano Federico:** Data curation. **Sevlever Gustavo:** Data curation. **Mezmezian Monica:** Data curation. **Marras Connie:** Writing – review & editing, Methodology, Investigation, Data curation, Conceptualization. **Gershanik Oscar:** Writing – review & editing, Investigation, Data curation, Conceptualization. **Chade Anabel:** Data curation. **Pardo Carolina:** Data curation. **Rellan Florencia:** Data curation. **Campastri Ana:** Data curation. **Couto Blas:** Writing – original draft, Supervision, Methodology, Investigation, Funding acquisition, Formal analysis, Data curation, Conceptualization.

## Declaration of competing interest

The authors declare the following financial interests/personal relationships which may be considered as potential competing interests: Couto, Blas reports financial support was provided by Curepsp. Couto, Blas reports financial support was provided by Alzheimer’s Association. If there are other authors, they declare that they have no known competing financial interests or personal relationships that could have appeared to influence the work reported in this paper.
